# Impact of the COVID-19 Pandemic on Student and Resident Teaching and Training in Surgical Oncology

**DOI:** 10.3390/jcm9113431

**Published:** 2020-10-26

**Authors:** Hans-Michael Hau, Jürgen Weitz, Ulrich Bork

**Affiliations:** Department of Visceral, Thoracic and Vascular Surgery, University Hospital and Faculty of Medicine Carl Gustav Carus, Technische Universität Dresden, 01307 Dresden, Germany; juergen.weitz@uniklinikum-dresden.de (J.W.); ulrich.bork@uniklinikum-dresden.de (U.B.)

**Keywords:** coronavirus, COVID 19, surgical oncology, surgical training and teaching, surgical education, E-learning

## Abstract

The COVID-19 pandemic has tremendously changed private and professional interactions and behaviors worldwide. The effects of this pandemic and the actions taken have changed our healthcare systems, which consequently has affected medical education and surgical training. In the face of constant disruptions of surgical education and training during this pandemic outbreak, structured and innovative concepts and adapted educational curricula are important to ensure a high quality of medical treatment. While efforts were undertaken to prevent viral spreading, it is important to analyze and assess the effects of this crisis on medical education, surgical training and teaching at large and certainly in the field of surgical oncology. Against this background, in this paper we introduce practical and creative recommendations for the continuity of students’ and residents’ medical and surgical training and teaching. This includes virtual educational curricula, skills development classes, video-based feedback and simulation in the specialty field of surgical oncology. In conclusion, the effects of COVID 19 on Surgical Training and Teaching, certainly in the field of Surgical Oncology, are challenging.

## 1. Introduction

Since the first COVID-19 case was detected in Wuhan, China, in late December 2019, this pandemic has affected virtually all countries across all continents and changed everyday life throughout the world [1]. This was the first time that the effects of a pandemic outbreak could be observed through all global health care systems within a very short time frame. It is likely that COVID-19 will not be the last global healthcare emergency we will encounter in our career as healthcare professionals. In this unprecedented scenario, healthcare systems around the world have rapidly begun to optimize and stretch their resources to effectively cope with this situation.

Although the focus of public health measurements has been to care for patients and communities, the emergence of severe acute respiratory syndrome SARS-COV2 has also invariably affected medical professionals in their academic and professional development and training from the undergraduate level to postgraduate and advanced medical education and disrupted all forms of medical training and teaching [2,3].

With regard to the field of surgical oncology, basic clinical and surgical principles and theoretical knowledge (clinical management of surgical issues, surgical knot tying, Virtual Reality Training, etc.) are most important for medical students and trainees/young residents, whereas hands-on training, (virtual) simulator training, skills development and advanced training in interdisciplinary concepts are important for older residents and surgeons.

In the setting of expert level, the advanced skills and interdisciplinary exchange with experienced specialists to master new concepts and surgical techniques is the most important part of surgical oncology training.

However, the current COVID-19 pandemic has a dramatic effect on these aspects and several problems and challenges exist for surgical education and training in this specialized field (Table 1).

In the setting of medical students, the consequences of the global COVID-19 spread for the medical education system are severe [4,5]. As a result of the pandemic, universities were closed, clinical rotations for undergraduates with patient contact, elective opportunities and clinical examination classes were suspended immediately and many traditional small group formats, lectures and face-to-face seminars were restructured or postponed [6].

These, together with a shortage of personal protective equipment and coronavirus tests, have contributed to the fact that medical students were moved away from undergraduate training, practical education and patient contacts. As a result, the optimization of practical skills as well as several non-technical skills are not possible, and the students got limited feedback or supervision of the tutors (Table 1).

In addition, many hospitals began to restrict, cancel or postpone all nonessential (oncological) operations and procedures as well as outpatient visits and clinics to protect vulnerable patient populations [7,8]. Further to prevent disease spread, some hospitals even asked nonessential medical staff to stay home [3,9]. Travel restrictions for business and educational purposes were introduced for medical staff, and almost all continuing medical education was cancelled or postponed [7].

Therefore, the impact of the COVID-19 pandemic on surgical care, education, training and teaching has been enormous [10,11]. The possible negative impact on the surgical skills of residents who have had no practical training experience for a period of time remains a challenge [7]. For trainees/residents in surgical specialties, canceled elective surgeries will inevitably affect their hands-on surgical experience, skills and case logs and therefore significantly disrupt their residency training [7,12]. Furthermore, elective operations are ones that residents usually performed with minimal supervision, whereas more complex and urgent are mostly performed by experienced residents. Regulations often permitting only essential personnel in the operating room further reduce intraoperative experience and training opportunities. Hands-on courses and cadaver dissection are prohibited, where training of practical and technical skills is possible. However, surgeons should have the opportunity to regularly train in practical skills—especially in the increasingly complex field of surgical oncology [4]. Clinical teams tend to be smaller with a fraction of residents staying at home in order to ensure availability of back-up personnel. Case discussions and department meetings, conferences, etc. are cancelled due to social distancing and staff availability concerns. This together further reduces the opportunity for continuous education, training and mentoring.

Today, surgical oncology is embedded in an interdisciplinary treatment setting. Thus, the introduction of multimodal therapy concepts, advances in perioperative medicine and new and modern resection procedures have led to significant advances in the treatment of solid tumors in recent years. Therefore, an interdisciplinary exchange with specialists to get to know new surgical concepts, new research results and surgical techniques are important for advanced surgeons. However, in case of the COVID-19 crisis, these traditional small group surgical training courses are cancelled and practical skill demonstration with in-person attendance and hands-on training are difficult to realize.

These unfathomable circumstances require creativity, flexibility and innovations. The profound effects of coronavirus disease may forever change how current and future physicians are educated and trained. Further major effects should be examined to focus on new models for delivering surgical education, teaching and training [11,13].

This begins with the training of the next generation of doctors through special clinical traineeships and internships and goes as far as to affect specialized expert training modules offered in comprehensive cancer centers (CCC) [4,14].

The following article is intended to provide insights into the various training and continuing education activities in a complex area of surgery—the field of surgical oncology.

This means that adequate training, further training and continuing education—starting with the next generation of doctors and extending to experienced colleagues—has become indispensable.

A special focus of the article lies in new possibilities and alternative modalities with potential advantages and drawbacks for delivering education, training and teaching both for surgical residents and medical students such as advanced surgeons during the COVID-19 pandemic.

## 2. Main Text

### 2.1. Introduction to Surgical Oncology and Oncological Surgery

The multidisciplinary field of surgical oncology is a perfect example of a complex and modern field of medicine where profound knowledge and practice skills are required to deliver good care. The terms surgical oncology and oncological surgery can be distinguished: a recent position paper defined surgical oncology as follows [15]:

- Participation in interdisciplinary working groups (Tumor Board, Tumor Center).
- Participation and implementation of pre-, intra- and postoperative therapy concepts.
- Participation in multimodal therapy concepts, together with the respective professional representatives of the other participating subjects.
- Tumor diagnostics and staging.
- Implementation of palliative treatment measures.
- Tumor aftercare including the scientific evaluation of the data.
- Participation in oncological rehabilitation and psycho-oncological care.
- Participation in cancer registries.
- Participation in the treatment of patients with hereditary tumor diseases.
- Oncological basic and clinical research (studies).
- Participation in the education, further education and training of medical professionals and the general public.

Surgical oncology, as an independent discipline, addresses considerably more content than the surgical techniques and surgical treatments of patients alone [16]. This aspect, called oncological surgery, is only one part of the field of surgical oncology and describes adequate oncological surgical techniques, and it focuses on the basic principles of standard oncological operations, which were essentially described in 1949 by K. H. Bauer: the complete R0 resection of the tumor, recurrence or metastasis must be performed as an en bloc resection technique. The trauma- and contamination-free technique is essential, as is an adequate lymphadenectomy technique (adapted to the operation). The planning and execution of the operation within the framework of a multimedia therapy concept and the choice of a correct reconstruction technique are also part of this [15].

Surgical oncology and cancer treatment is complex with a range of urgencies for intervention, a large variety of potential treatment options and numerous treatment sequencing options [14,16]. Several papers presented the great challenges that oncologists are facing during the COVID-19 pandemic advising about an above 3-fold risk of infection in oncological patients [17].

Moreover, cancer patients are often older and have an exponential infectious risk related to an immunodeficiency state from cytotoxic chemotherapy and weakness deriving from multiple and potentially life-threatening comorbidities. These factors together converge to further intensify the cancer management and treatment options during the current COVID-19 pandemic. In this context, usual recommendations as already mentioned for minimization of COVID-19 infection consist of disposable personal protective equipment for workers and patients, social distancing in waiting rooms/wards, prohibition of accompany patients such minimization of time staying in the hospital rooms.

Special attention should be paid in reduction the hospital attendance through the delay of non-urgent counselling, adoption of preadmission telephone triage and implantation of telemedicine [18]. Beyond all these general precautions, in a geriatric setting, it is of importance to stratify patient´s individual risk due to cancer therapy and analyze patients benefits from therapy [19]. Herein, a complex evaluation of prognostic factors and scores of patients’ well-being/health (e.g., by application of Comprehensive Geriatric assessment (CGA)) such as of toxicity related to chemotherapy (e.g., by application of Chemotherapy Risk Assessment Scale for High-Age Patients (CRASH)) is—not only during this pandemic—essential to choose the best treatment with the lowest risk for this population [19].

### 2.2. Surgical Oncology for Medical Students

An up-to-date, modern training concept is a central element for the next generation of surgeons today. Currently, medical knowledge alone is no longer sufficient as the basis for medical proficiency [20]. Rather, one of the tasks of university teaching is no longer just the pure transfer of knowledge—so-called “teaching”—but rather the transfer of the ability to “learn”.

Competency-based medical education and training is seen as a central prerequisite for preparing medical students for the requirements of adequate medical care in the 21st century [21]. However, the novel coronavirus pandemic has greatly affected our medical education system, and the infrastructure of our education systems is being compromised as never before. Due to the highly infectious nature of SARS-COV-2, classic static teaching formats with face-to face interactions in small and large group settings (lectures, tutorials, etc.), laboratory sessions, simulations and/or any forms of patient contact in clinical clerkships and/or rotations have not been possible during the current phase of the pandemic [2,11]. As an important part of surgical oncology, medical students’ participation and/or observation of surgical oncological procedures in operating rooms have been suspended. Regulations of several hospitals permitted only essential personnel to preserve available personal protective equipment [22].

This has precluded students from being active team members as hospitals attempt to minimize nonessential staffing in clinical environments [2,11].

As a consequence, medical educators have rapidly tried to change their education programs and curricula by moving as much online as possible in a digitalized format. Therefore, e-learning and blended-learning, such as “flipping the classroom” concepts, are the new focuses for education and teaching, and not only in surgical oncology, during the COVID-19 pandemic [11,23,24].

Efforts within the last years and the time of this crisis have shown that this new learning forms considered to be efficient compared with classical, traditional teaching methods [11]. Potential advantages and drawbacks compared to the traditional learning activities for our described three groups that can be used for education and training in surgical oncology during COVID-19 are summarized in Table 2.

In this context, several e-learning resources are available including interactive electronic and virtual educational platforms, webinars, video- and teleconferencing applications, social media, surgical simulations apps, etc. that can be used to deliver lectures or tutorials remotely via handheld devices and laptops [2,3,11,25]. Medical students and residents can then log in at designated times for online lectures and discussions, meet each other “regularly” and learn from local lectures that can be facilitated in real time via teleconferencing applications (Zoom^®^, Webex^®^, etc.). These platforms allow lecturers to share and show slides in real time as well as record lectures for student participation at later times [2,11] (Table 2).

In addition to virtual online lectures, video- and teleconferencing can be used to demonstrate medical procedures or surgical techniques [2,11]. Platforms exist to explain clinical examinations online and develop differential diagnoses and improve clinical skills and reasoning. In small group formats that can be convened online in virtual team settings, patient cases and exciting topics can be discussed [10,26].

Last, medical students and residents should be encouraged to use online resources to facilitate their individual learning. Specifically, virtual surgical oncology-based programs present surgical videos and detailed steps of given operations, provide surgical anatomy reviews and contain sections for self-assessment and practice test questions [4,11].

However, amid this extraordinary emergency, challenges can be transformed in opportunities that means that benefits of these new learning activities with regard to the classical teaching and learning activities should be considered as an excellent way for education for progress. In detail, benefits with regard to the group of medical students range from the introduction of a new and innovative educational curricula, individual time management to increased availability of more interactive online surgical platforms and VR-simulators at the teaching hospitals (Table 2).

These different strategies were adopted in our center, for example, where, with regard to the special field of surgical oncology, learning-relevant surgical-oncological contents were presented in virtual learning platforms and applications (podcasts, video conferences, etc.).

The presentations of highly complex instructional films, online webinars or live surgeries were additionally integrated in our standardized surgical oncological curriculum and training program. In our opinion, this increased the knowledge of specific clinical oncological thinking and operative oncological understanding. In addition, online monitoring is currently planned where medical teachers in online discussions could further help students consolidate their learning. Feedback from teachers is invaluable and can be achieved with the use of online video communication services in which the teacher can utilize screensharing options to walk the students through relevant surgical oncological topics or surgical operative steps. The use of current virtual patient cases (prior real patient cases) covering different oncological topics and diseases can help to consolidate what has been learned.

However, online learning is not feasible and reasonable for all courses and aspects during surgical education and teaching and several drawbacks exist [26,27,28] (Table 2). Therefore, the core competence of a trained oncological surgeon is first and foremost his or her specialist knowledge and the practical skills that can be learned, as well as surgical intuition developed through experience [16]. The earlier practical and technical skills and abilities can be learned and practiced in the course of training, the easier it is to apply what has been learned in the clinic in dealing with patients [26,29].

At our center, for example, we have made it our goal to teach not only the principles and theoretical knowledge of surgical oncology but also the concrete practical techniques of oncological surgery.

Prior to COVID-19, medical students would be given the opportunity to learn a wide range of manual skills, in particular through special clinical traineeships and for final-year medical students during their surgical elective [10,26]. The spectrum ranges from examination techniques and interventions to complex surgical and even operative manual skills, in addition to the knowledge of anatomical-topographical and physiological associations, which are based on manual skills as well as the interaction of technical factors such as instruments and/or suture materials. In special practical training modalities, so-called skills labs, students are offered practical surgical management in a protected learning environment [30]. Through the use and application of phantoms/models, training dummies, laparoscopy/robotic simulators and/or mutual practice of students, individual medical skills can be learned and trained in peace and quiet, which leads to a significant increase in overall patient safety and decreased intraoperative errors [31].

To adhere to recommendations for social distancing, many group and box training simulation sessions have been canceled. However, times like this make us appreciate all that is special about surgical training and highlight the mere supplementary role of online and innovative technical resources in student and residency curricula. Currently, computer- and phone-based technologies are used to provide access to intraoperative video recordings, virtual reality (VR)-operating room simulations and other interactive surgical platforms [10,11,32].

Models and instruments, such as suturing kits with artificial tissues or portable fundamental laparoscopic box trainers, can be borrowed for technical practice [28,32].

Such widely available applications have the potential to satisfy and supplement the learning needs of young surgeons in education and to continue hands-on training in these difficult times [11].

### 2.3. Surgical Oncology for Residents and Fellows

Training and teaching surgical oncology for residents and fellows requires significant hands-on experiences, exposure to peri-and postoperative management of oncologic patients, rotating through different services with distinct patient populations and formal education sessions such as grand rounds and multidisciplinary tumor conferences [4,14,16].

The current COVID-19 pandemic has had a dramatic effect on these aspects of surgical oncology training and education.

With regard to COVID-19, some surgical oncological programs, e.g., in regions with a significant burden of COVID-19 cases, have seen a decline in nonurgent oncologic and other elective procedures due to concerns with COVID-19 exposures and PPE shortages [4,7,26].

In efforts to resolve these issues, surgical services and residency programs, especially in COVID-19 epicenters such as New York and/or Italy, were restructured [9,13]. This consisted of several measurements used to safely deploy the necessary amount of the resident workforce to support pandemic efforts while maintaining staffing for emergency surgical care, limiting unnecessary exposure of residents to infection risk, effectively placing residents in critical care units and maintaining surgical education and board eligibility for the training program as a whole [13].

A further easy and effective possibility to reduce and prevent possible hospital contagion among hospital workers and patients could be done by telephonic triage and telemedicine

With the help of an exhaustive questionnaire which was administered via telephone call by a trained physician to all patients referring to a surgical division for an elective surgical intervention (three days before admission)/ for outpatient visits in-hospital infections and further collapse of the health care systems could be reduced [18].

In cases of symptoms, return from a high risk location or contact or cohabitation with positive COVID cases or suspicious ones, the patients were asked to procrastinate the hospital admission for almost 14 days.

Nevertheless, a crisis such as this provides opportunities for medical teachers and educators to adopt new and innovative learning concepts to maintain the quality of medical training and teaching (Table 2) [2]. With regard to the field of surgical oncology, it is important to find ways and platforms to continue education and surgical training.

In this context, specialized and structured surgical oncology training and education curricula can feature helpful tools for delivering knowledge and education in this particular medical field [4].

As is known, the therapy of malignant tumors has made great progress in all disciplines involved in recent years but is becoming increasingly complex. Therefore, a thoroughly planned and conducted educational curriculum for residents and fellows is essential [14,16]. Specialized training and educational modules can ideally be prearranged by centralized organizations, such as schools of surgical oncology. Our center has founded the Dresden School of Surgical Oncology (DSSO), for example, to offer structured in-house courses for residents, fellows and advanced specialists in the field of surgical oncology. The offered courses feature tailored approaches developed for different types of experience levels. In this context, organ-specific (stomach/esophagus, colon/rectum, pancreas, liver) classes can be offered. The different viscero-oncological therapy indications, concepts, intentions and objectives are explained, as are surgical techniques and training of skills in practice sessions. The focus is also on multidisciplinary treatment concepts, neoadjuvant and adjuvant treatment concepts and palliative therapy options, which all represent learning contents of surgical oncology. The DSSO is also embedded in the comprehensive cancer center setting of the National Cancer Center (NCT) in Germany. Precise knowledge of the tumor disease, the options of multimodal therapy and broad surgical knowledge are required for the surgical oncologist. A further focus of these courses is the explanation of the basic principles of oncological surgery. The indication for surgery, the selection of the various resection procedures and possibilities as well as the technique of surgical treatment are discussed in detail. For example, the principles of complete mesocolic excision (CME) with a radical no-touch technique and monoblock resection of the tumor-bearing colon, central vascular ligation and anatomy-oriented lymphadenectomy with radical resection of the mesocolon are discussed theoretically on the basis of colon carcinoma.

Prior to COVID-19, these courses were held in face-to-face, physical-based activities via small group tutorials, and the participants of the courses could then consolidate their theoretical knowledge in practice by means of observations of the techniques in the operating theater and by face-to-face video demonstrations. However, during the pandemic phase of COVID-19, all surgical-based education and training curricula had to be restructured and reorganized. Analogous to the medical students’ educational curricula, new technologies and virtual learning modalities were used at our center to forge the future of resident education and training in this field and to better prepare them when they return to their clinical rotations [7,11,26,27,28].

With the help of video-based educational tutorials such as computer-based surgical platforms (e.g., own intraoperative surgical oncological videos, open-access video-based online platforms (American College of Surgeons, Websurg^®^, deutsche Gesellschaft für Chirurgie (DGCH), Journal of Medical Insight (JOMI)) detailed steps and aspects of the most important surgical oncological operations could be illustrated, learned and repeated [27,28]. These resources instruct residents on proper surgical indications, preoperative workup and operative anatomy and techniques. Structured virtual live or recorded lectures in online classrooms or safe virtual classroom settings, analog and digital handoffs and podcasts could help to solidify the learned contents [11,24,27,28]. This has allowed for flexible learning during traditional residency schedules and clinical activities. Furthermore, active discussions with surgical teachers are performed via these virtual learning modalities for supervision and skills improvement. Further benefits of these new learning modalities exist in the introduction of new structured curricula and training pathways, larger pools of experienced tutors and geographical flexibility (Table 2).

Although virtual learning can support certain aspects of surgical education and training, a challenge remains for surgical centers and institutions, as virtual learning is not convenient for all aspects of learning and training for residents in surgical oncology, and several drawbacks are evident [26].

With the COVID-19 pandemic, many surgical residency programs are subject to continuous interruptions [13,23]. The cancellation of elective operations leads to decreased operating time for surgical residents and therefore a decrease in training opportunities to learn surgical practical skills during this time [7,26]. Therefore, intraoperative practice and experience have declined considerably.

During the current restrictions, exposure of residents to conventional teaching cases has been limited. Therefore, residents have less time to complete procedural quotas and fewer opportunities to gain familiarity with cases and procedures [26]. In this context, other programs and tools are mandatory to deliver necessary educational and training experiences.

Surgical simulation has become increasingly important in recent years and already represents an important part of residents’ practical training and continuing education of surgical skills under supervision [27,33]. Through regular training and the use of surgical simulators, basic skills as well as complex surgeries can be learned [33].

Several training models have been proposed for learning/teaching surgical laparoscopic skills, including box trainers (BT) and virtual reality simulators (VRS) [32]. While VRSs are immobile, expensive and equipment not readily available for use at home, BTs are inexpensive and can be constructed from household items [34,35]. Additionally, many residency programs support the use of home laparoscopy box trainers, which may be a suitable replacement for virtual reality simulators, which are only available at hospitals [36]. Several groups have described good results with improving surgical performance using BTs at home [37]. However, when a VRS is available at a hospital, it should be used for residency training because it provides cognitive and coaching feedback not included in a BT [32]. In addition, programs can be introduced to directly fulfill procedural quotas and to make residents familiar with advanced operations and other specialties [26].

As it already known, operating surgeon technical skill is directly associated with postoperative outcomes, yet few options exist to support learning and practicing surgeons in improving technical skills. In this context, surgical coaching, consisting of debriefing, video review and feedback has emerged as beneficial tool to improve technical skills and address variation in clinical outcomes, both in fellows such as practicing/advanced doctors [38,39].

To further imitate the real-world surgical environment, written or verbal feedback can be provided remotely, either through faculty review of uploaded recordings or hosted video chat sessions, in which experienced surgeons review operative techniques and discuss procedural nuances in surgical videos [27]. Other teaching methods include residents, who have to solve different tasks during simulator training for faculty feedback in real time. In-hospital residents could use the free time to individually practice on simulation labs and robotic trainers. Residents can capitalize on near-peer learning by receiving feedback from their fellows standing 1.5 m away on their technical skill performance [25,27].

Another excellent possibility—especially in these times—to improve surgical technique could be video-based coaching with the help of a video-shared online surgical platform (VISTA).

Recently, Schlick et al. reported their experiences with this technique to provide a safe environment for practicing surgeons to improve existing techniques and learn new surgical skills through structured peer feedback [38].

However, in addition to surgical training, theoretical and oncological knowledge and experience in a broad range of different cancer subtypes is of uttermost importance in surgical oncology [14]. Well-trained surgical oncologists must be familiar with the full spectrum of therapeutic options for a large variety of malignancies, including their use in the neo- and adjuvant settings. In our clinic, we have a structured educational curriculum (Table 2) with internal and external advanced training courses, teaching ward rounds and different journal clubs, which take place several times a week, among other things with different disciplines such as pathology or radiology. Furthermore, the indications of all operations, especially oncologic operations, are discussed at a daily conference.

In addition, case discussions and morbidity and mortality conferences are held once a week to address critical issues and improve clinically safe processes. Another possibility of developing a deeper understanding of certain topics and learning new knowledge consists of self-study and self-directed learning for our residents and fellows.

Only through knowledge of the results from recent clinical trials and data from other oncologically active departments can one’s own therapeutic tools be evaluated, classified and weighted in a multidisciplinary treatment setting [14,16]. In particular, sound oncological knowledge in all areas of cancer medicine allows for the best possible oncological treatment option for the patient to be determined in a collegial exchange, for example, in tumor boards.

During the COVID-19 pandemic, all these aspects of clinical life and residency education and training take place online via electronic platforms and programs (Table 2).

### 2.4. Surgical Oncology for Advanced Doctors

For senior physicians and chief physicians who are advanced in their professional lives, innovative and modern techniques of oncological surgery, as well as new interdisciplinary treatment methods and their implementation, are the focus of discussion [14,16]. These can be taught in small-group advanced training courses, for example. These concepts are ideally not designed as classroom teaching concepts. A basic principle of these teaching modalities should be open dialogue, in which it is not uncommon for lecturers not only to impart knowledge but also to gain knowledge themselves through discussion with experienced course participants. This applies in particular to the reality of surgical care and the implementation of certain techniques [16,40].

One example for these kinds of courses is the advanced training classes from the DSSO, which began its work in 2013 with a two-day course on modern surgical procedures for rectal cancer. Experienced lecturers, including Sir Richard (Bill) Heald, the inventor of total mesorectal excision in rectal cancer (TME), taught the theoretical content. Practical contents were conveyed through observation and live transmissions of operations from various operating theatres. This concept, which combines theory and practical examples through live surgery as well as video demonstrations, has proven its worth. Consistent small group courses with a maximum of eight participants enable a particularly close dialogue with the course participants.

The latest research results, for example, on tumor cell organoids and their role in the investigation of neoadjuvant treatment concepts, can also be directly incorporated into the course content [41].

Especially in the case of advanced and complex diseases, individual treatment strategies that are coordinated in a multidisciplinary manner are all the more important and are carried out in case discussions.

Since 2013, 4–6 DSSO courses have been held annually in the fields of rectal carcinoma, pancreatic carcinoma, stomach carcinoma, liver metastasis surgery and a basic course in surgical oncology. To date, more than 200 surgeons from all over Germany have taken part in DSSO courses.

The courses are continuously evaluated by the participants and lecturers and regularly receive excellent feedback, especially due to the small group sizes and the constructive-critical learning atmosphere, which is perceived as consistently pleasant.

However, in the current COVID-19 pandemic, the in-person attendance of surgical training courses is difficult, and a restructuring of this event was performed at our center.

Therefore, the course programs were shifted to online modules with the use of video- and teleconferencing platforms, podcasts, webinars or virtual surgical platforms.

For practical skill demonstration and hands-on experiences, our skills lab was rebuilt. Herein, we now had the possibility of appropriate distances to undergo box training and VR simulator training.

Furthermore, in times where in-personal meetings and face-to-face activities are difficult, video-based coaching could be a helpful tool in surgical education [38,39].

Herein, structed operative video-based feedback and review for surgical technique improvement via an online surgical platform (VISTA) is already mentioned a relatively new concept to support practicing, advanced doctors [38].

As a part of a structured surgical oncological operative training course (e.g., DSSO course) and/or surgical oncological curriculum for advanced doctors, VISTA could include the improvement of operative technique and performance by focusing on skills directly, development of introspection skills and fostering of an ability to identify areas for improvement within the operating surgeon themselves, even with a dedicated expert coaches input [38].

## 3. Conclusions

Medical crises such as the COVID-19 pandemic will undeniably affect medical students and residents’ training and education in surgery at large and certainly in the field of surgical oncology.

Surgical oncology today is a highly complex field of expertise embedded in the multimodal therapeutic concepts of oncological diseases.

Prevention of the COVID-19 contagion, such as managing of infection in older patients with cancer, is extremely challenging and requires multidisciplinary approach. An identification of subsets of patients which benefit from cancer therapy could be helpful, especially in for older patients with cancer.

Furthermore, training and further education in surgical oncology begins during medical studies and continues throughout professional life.

The rapid embrace of web-based platforms and virtual learning resources for residents and students could be helpful for the continuation of educational curricula not only in surgical oncology.

Considering these circumstances, significant advances and technological developments in surgical simulation and virtual reality will leave us far more prepared for any similar crisis on the horizon.

Ultimately, the challenges created by COVID-19 will be overcome through novel solutions that can empower the next generations of surgical oncologists.

## Figures and Tables

**Table 1:** Problems and Challenges through COVID-19 for Surgical Teaching and Education.

- Disruption of clinical clerkship (especially practical skills training)/lectures observations/elective opportunities → Limited feedback, no optimization of practical and technical skills, no supervising of learning effects. - Cancellation of elective operations and disruption of elective operating room attendance → no optimization of practical and technical skills, preserving available personal protective equipment, lack of intraoperative experience, no supervising and feedback, no mentoring. - Cancellation of advanced surgical training courses, no-in person attendance, missing collegial exchange. - Concern with COVID-19 exposures. - Personal protective equipment shortage. - Lacking interpersonal communications, social distancing, home isolation. - No on-campus activity. - Disruption of surgical residency education and training. - Self-motivation required. - Mental health problems, anxiety. - Time management skills required. - More theoretical rather than practical surgical skill learning. - Availability of back-up personnel.

**Table 2:** Learning contents and new leaning modalities with benefits and drawbacks for education and training for surgical oncology during the COVID-19 crisis.

Groups	Learning Contents in Surgical Oncology	Traditional Learning Forms	New Learning Forms	Advantages and Drawbacks of New Learning Forms
**Students**	Theoretical and clinical surgical education	- Oral main lectures, frontal lessons. - Small group lessons and seminars. - Surgical clinical practice and practical training (“bed-side teaching”, direct patient contact). - Problem-learned interactions. - Group-learning. - Local exams. - Clinical skill sessions and clerkship.	- E-learning and blended learning modalities. - Virtual didactics (online lectures, webinars, podcasts). Video and phone-based conferencing platforms (Zoom^®^, Skype^®^) (Live case presentations, surgical case discussions) - Online learning tools (“Flipped classroom concepts”). - Computer based online surgical simulation platforms (Virtual patients, anatomy atlases, diagnoses quizzes, virtual visiting grand rounds, physiology and pathology courses, etc.). - Social media (Twitter). - Self- directed learning. - (Textbooks, described virtual educations modules).	Advantages: - Introduction of structured education curricula and strategies. - No geospatial and temporal limitations. - Repeatability and Reliability of online learning tools (lectures, simulation platforms). - Larger pool of tutors. - Individual time management. - Self-placed leaning. Drawbacks: - No face-to-face teaching/ no personal interaction. - No optimization of practical and technical skills, no direct supervising of learning effects. - No training of real life or complicated situations. - Loss of patient contact. - Self-motivation required.More theoretical rather than practical surgical skill learning. - Adequate Assessment of skills and knowledge.
	Surgical practical education	- Physical examination courses. - Objective structured clinical examination (OSCE) courses. - In-person anatomy dissections. - Participation in operating room. - Skills laboratory exercises (Knot and suture, Pelvi- and box training, ultrasonography courses). - Surgical simulator training (virtual reality simulator and/or laparoscopic box training, cadaver training, etc.).	- Skill-based virtual learning tools. - Operative video- based education (Own surgical instructional videos, online video library of American College of Surgeons and Deutsche Gesellschaft für Chirurgie (DGCH)), YouTube^®^, WebSurg^®^). - Computer-based education, surgical platforms (JOMI, Surgery Squad, etc.), virtual operation room, simulations virtual bootcamps and classrooms surgical online, training and skills workshops, skills laboratories. - Surgical simulation-based education, laparoscopic box training, virtual reality simulator training. - Homemade surgical simulation models. - Videogaming.	Advantages: - Introduction of structured training pathways. - Online learning contents→ Repeatability, Reliability. - Availability of more interactive surgical platforms/VR-Operating room simulators/intra-operative video recordings. - Increase of operative technical proficiency and more time of training. Drawbacks: - Virtual Simulator training: Technical limitations (screen resolution). Running costs. No haptic feedback. - No hands-on experience. - No cadaver dissection. - No training of real life or complicated situations. - Less time spend in operating room→ lack of intraoperative experience and practical skills. - Lack of surgical exposure→ loss of recruitment of young surgeons. - Availability of training options (Simulators).
**Residents**	Surgical practical education	- Participation and performance of practical surgical training in operating room, e.g., elective operations in surgical oncology (hemicolectomy right, etc.). - Skills laboratory exercises (knot and suture, Pelvi- and box training, ultrasonography courses). - Surgical simulator training (virtual reality simulator and/or laparoscopic box training, cadaver training, etc.).	- Operative video-based education (own surgical videos, online video library of American College of Surgeons and Deutsche Gesellschaft für Chirurgie (DGCH)), YouTube^®^, WebSurg^®^). - Virtual didactics. - Virtual lectures. - Flipped classroom concepts. - Step-by-step tutorials. - Online surgical courses. - Computer-based education. - Surgical platforms (JOMI, Surgery Squad, etc.). - virtual operation room simulations. - surgical online training. - skills laboratories. - Surgical simulation-based education. - Laparoscopic box training. - Virtual reality simulator training. - Homemade surgical simulation models. - Videogaming.	Advantages: - Online learning contents and surgical kits→ Repeatability, Reliability. - Introduction of structured training pathways. - Individual Time management. - Increase of technical proficiency. Drawbacks: - Decrease in the availability and acceptance of practicing technical skills on patients. - Virtual Simulator training: Technical limitations (screen resolution). Running costs. No haptic feedback - No hands-on experience. - No cadaver dissection. - No training of rea life or complicated situations - Less time spend in operating room→ lack of intraoperative experience. - Loss of surgical practice and experience.
	Clinical education and training	Grand rounds Assigned bedside rounds Practical skills	- Virtual platforms. - Virtual didactics (lectures, webinars, podcasts). - Video and phone-based conferencing platforms (Zoom^®^, Skype^®^). - Live interactive virtual rounds. - Computer-based platforms (Web Surg, Teach Me Surgery, Anatomy Atlas, etc.).	Advantages: - Larger pool of tutors - Online learning contents and surgical kits→ Repeatability, Reliability. - Introduction of structured training pathways. - Individual Time management. Drawbacks: - No face-to-face teaching/no personal interaction. - no optimization of practical and technical skills, no direct supervising of learning effects. - No training of real life or complicated situations. - Self-motivation required. - More theoretical rather than practical surgical skill learning. - Decrease in the availability and acceptance of practicing technical skills on patients.
	Ambulatory/outpatient clinical setting	In-person clinical visits and direct patient contact	- Video- and phone-based conferencing platforms (Zoom^®^, Skype^®^). - Telehealth, virtual consults. - Telemedecine. - Working with masks. - Self-directed/independent learning (textbooks, online review articles, described virtual educational modules).	Advantages: - Prevent of contagion. - Cost-effectiveness. Drawbacks: - No personal Patient-doctor relationship. - Legal problems (surgical enlightenment).
	Standard educational conferences (multidisciplinary tumor board meeting, journal club, operative indication conferences, morbidity and mortality conferences, specific oncological case conferences, congresses)	Face-to-face interactions, interpersonal communications, small group formats	- Video- and phone-based conferencing platforms (Zoom^®^, Skype^®^). - Online chat. - Self-directed/independent learning (textbooks, online review-articles, described virtual educational modules).	Advantages: - Less time spend travelling. - Temporal Flexibility. - No geographical barriers. - More offers. Drawbacks: - No face-to-face and personal interaction. - Scientific and quality standard lacking. - Less feedback and direct supervising of learning effects.
	Research	Personally working in laboratories	- Virtual research meetings by video-based conferencing platforms (Zoom^®^, Skype^®^)	Advantages: - Individual Time Management. - Self-directed Working. - Introduction of new educational research curricula. - Geographical barriers are removed. Drawbacks: - No face-to-face and personal interaction. - Less feedback and direct supervising of learning and training effects. - Limited possibilities to continue basic science and laboratory research. - National policy restrictions.
**Senior physicians**	Advanced surgical skills	Advanced clinical training courses	- Computer-based surgical platforms (e.g., own intraoperative surgical oncological videos, open-access video-based online platforms (American College of Surgeons, Websurg^®^, DGCH, Journal of Medical Insight (JOMI)) - Virtual didactics (webinars, live and recorded lectures, podcasts by Zoom^®^, Skype^®^, etc.) - Self/independent learning (review materials, textbooks…) - Surgical simulation models (robotic/laparoscopic training/cadaver training…)	Advantages: - Less time spend travelling - Introduction of structured education curricula and structured training pathways - Increased availability of online platforms/ intraoperative videos for advanced doctors - More time for self-directed learning/surgical training - Global connection and larger pool of experienced tutors Drawbacks: - No face-to-face activities/ no personal interaction - Less feedback possibilities from experienced colleagues and supervision - No cadaver dissection and hands-on experience
	Advanced knowledge of complex cases/ New research results	Conferences, clinical meetings (Face-to-face interactions, interpersonal communications)	- See above	See above
